# 3D Radiation Therapy Boost Improves the Outcome of Whole Brain Radiation Therapy Treated RPA II Patients with One or Two Brain Metastases

**DOI:** 10.3390/ijms15057554

**Published:** 2014-05-02

**Authors:** Delphine Antoni, Jean-Baptiste Clavier, Marius Pop, Catherine Schumacher, François Lefebvre, Georges Noël

**Affiliations:** 1 Radiotherapy Department, Centre de Lutte Contre le Cancer Paul Strauss, 3, rue de la Porte de L’Hôpital, 67065 Strasbourg Cedex, France; E-Mails: JClavier@strasbourg.unicancer.fr (J.-B.C.); MPop@strasbourg.unicancer.fr (M.P.); CSchumacher@strasbourg.unicancer.fr (C.S.); GNoel@strasbourg.unicancer.fr (G.N.); 2 Laboratory of Biostatistics, Faculté de Médecine, 4, rue Kirschleger, 67085 Strasbourg Cedex, France; E-Mail: Francois.lefebvre@chru-strasbourg.fr

**Keywords:** brain metastases, whole brain radiotherapy, radiation boost

## Abstract

**Purpose:**

to evaluate the role of whole brain radiotherapy (WBRT) and radiation boost (RB) for 208 patients recursive partitioning analysis (RPA) II with 1 or 2 brain metastases (BM) at a single institution.

**Methods and Materials:**

the dose of WBRT was 30 Gy (10 fractions of 3 Gy). One hundred thirty-two patients (63.5%) benefited from RB of 9 Gy in 3 fractions of 3 Gy at the metastatic site. Patients had 1 or 2 BM in 122 (58.7%) and 86 cases (41.3%), respectively.

**Results:**

patients with one or two metastases had similar survival (4.6 and 5.1 months, respectively) (*p* = 0.4). Median overall survival (OS) for patients treated with WBRT and RB, and with WBRT alone was 5.9 and 3.7 months, respectively (*p* = 0.03). The 6-, 12- and 24-month OS rates after WBRT and RB were 48.5%, 25% and 10.6%, respectively, while WBRT alone resulted in OS rates of 34%, 22.4% and 3.2%, respectively (*p* = 0.03). After WBRT and RB, the 6-, 12- and 24-month local control rates were 92%, 82% and 67%, respectively, while they were 81.2%, 75% and 37.5%, respectively, after WBRT alone (*p* = 0.03). The 6-, 12- and 24-month brain control rates after WBRT and RB were 88.7%, 75.8% and 62%, respectively, and after WBRT alone they were 78.5%, 59% and 37.7%, respectively (*p* = 0.03).

**Conclusion:**

additional boost delivered with 3D conformal radiotherapy improves local and brain control rates significantly as well as overall survival for RPA II patients with 1 or 2 unresectable BM.

## 1. Introduction

Brain metastases (BM) remain a clinical and therapeutic challenge, and the optimal treatment remains a controversial subject. The incidence of BM increased from 7 per 100,000 to 14 per 100,000 between 1987 and 2006 [1,2]. Whole brain radiation therapy (WBRT) has been the most common treatment for years, but this standard of care is now becoming inappropriate for many patients due to the development of new treatment modalities like radiosurgery and because the outcomes of patients with BM depend on the tumor type [3–5]. WBRT alone is the standard treatment for patients with multiple BM. Patients with a few BM and a controlled primary tumor may benefit from local treatment such as surgery or radiosurgery in addition to WBRT [6–8]. The EORTC 22952-26001 study showed that combined therapy (surgery or radiosurgery and adjuvant WBRT) compared to surgery or radiosurgery alone improved the 2-year local control rate (*p* = 0.04) and the brain control rate (*p* = 0.02) [9]. The purpose of this study was to evaluate the role of a 3D-radiation therapy (RT) boost at the metastatic site combined with WBRT in the management of 1 or 2 BM in recursive partitioning analysis (RPA) II patients.

## 2. Results

There were 153 men (73.6%) and 55 females (26.4%) in this study. Two hundred and eight patients with a median age of 64 years (20.9–86.2) at diagnosis were treated with whole brain radiotherapy; 132 patients (63.5%) received WBRT with a radiation boost at the metastatic site, and 76 patients (36.5%) received WBRT alone. Diagnosis was established by contrast CT scan for 135 patients (64.9%), MRI for 25 patients (12%) or both CT scan and MRI for 47 patients (22.6%); we did not have data for one patient. There was an equal proportion of patients diagnosed by CT scan, MRI or both in each cohort. BM were located in the cerebral hemispheres (76.9%), in the cerebellum (12%) or at both of these sites (11.1%). Primary tumors were defined as lung tumors (*n* = 137, 65.9%), breast tumors (*n* = 18, 8.6%), gastrointestinal tumors (GI) (*n* = 16, 7.7%), melanomas (*n* = 10, 4.8%), and kidney tumors (*n* = 7, 3.4%) or other sites (*n* = 20, 9.6%). Patients had 1 BM in 122 cases (58.7%) or 2 BM in 86 cases (41.3%). The two treatment groups were comparable except for the control of the primary tumor; patients treated with WBRT and a boost had a better-controlled primary tumor than those treated with WBRT alone (*p* = 0.04).

### 2.1. Median Survival after Diagnosis and Treatment

Overall median patient follow-up time was 4.8 months (0–56.4 months), while median follow-up time of surviving patients was 30.3 months (12.4–56.4 months). Eleven patients (5.3%) were alive at the time of analysis, and 197 (94.7%) were deceased. The proportion of patients that had died was similar between the two treatment groups. Seven WBRT and boost treated patients (5.3%) were alive at the time of analysis, while 125 patients did not survive (94.7%). In the group treated with WBRT alone, 4 patients were alive at the time of analysis (5.3%) and 72 patients were deceased (94.7%). When considering both treatment groups, neurological death occurred in 23 patients (11.7%), while non-neurological death occurred in 103 patients (52.3%); the cause of death in 71 patients (36%) was unknown or the information not available. In patients treated with WBRT and a boost, 10 patients died of neurological causes (8.0%), 64 patients (51.2%) died of non-neurological causes, and 51 patients (40.8%) died of unknown causes. In patients treated with WBRT alone, thirteen deaths (18.0%) were attributed to neurological causes, 39 patients (54.2%) died of non-neurological causes, and 20 patients (27.8%) died of unknown causes. The difference between the two treatment groups was not significant.

**Figure 1.**
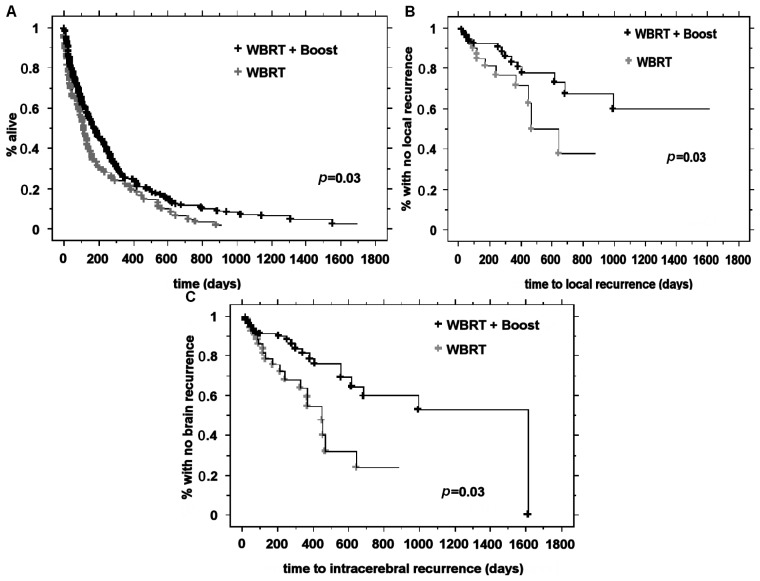
Comparison of whole brain radiation therapy (WBRT) alone and WBRT plus boost in (**A**) overall survival; (**B**) local control and (**C**) brain free-relapse survival.

Patients with one or two metastases had similar median survival times (4.6 and 5.1 months, respectively) (*p* = 0.4). Median survival time for patients treated with WBRT and a radiation boost at the metastatic site was 5.9 months, and median survival time was 3.7 months for patients who did not receive a boost (*p* = 0.03) (Figure 1A). The 6-, 12-, 18- and 24-month overall survival (OS) rates after WBRT and a radiation boost were 48.5%, 25%, 17.4% and 10.6%, respectively, and after WBRT alone, they were 34%, 22.4%, 12% and 3.2%, respectively (*p* = 0.03) (Table 1).

For patients treated with WBRT and a boost, univariate analysis demonstrated that improved OS was significantly associated with gender (*p* < 0.0001), type of primary tumor (*p* < 0.0001) and high Karnofsky Performance Status (KPS) (*p* = 0.02). Multivariate analysis demonstrated that favorable and significant prognostic factors for OS were gender (female) (HR 0.54 CI 95% 0.1–0.92, *p* = 0.02), breast cancer *vs.* other primary cancer subtype (HR 0.3 CI 95% 0.11–0.77, *p* = 0.03) and high KPS (HR 0.66 CI 95% 0.44–0.98, *p* = 0.04).

For patients treated with WBRT alone, univariate analysis demonstrated that improved OS was significantly associated with primary tumor type (*p* = 0.01) and high KPS (*p* = 0.05). Multivariate analysis demonstrated that OS was significantly associated with high KPS (HR 0.55 CI 95% 0.32–0.93, *p* = 0.02) and primary tumor type (*p* = 0.02); lung cancer, melanoma and gastrointestinal cancer were unfavorable prognostic indicators (HR 2.5 CI 95% 1.01–6.44, *p* = 0.04; HR 6.3 CI 95% 1.8–21.9, *p* = 0.003; HR 6.9 CI 95% 1.78–27.3, *p* = 0.005, respectively). Patients with breast cancer had an improved outcome compared to those with lung cancer, renal cell cancer, melanoma, gastrointestinal cancer, or other types of cancer: median survival times were 16.5, 5.6, 3.2, 2.6, 1.8 and 4.2 months, respectively (*p* < 0.0001).

Patients classified as RPA II for breast cancer and treated with WBRT and a boost had significantly improved overall survival compared to those who did not receive a boost: 26.6 *vs.* 9.1 months, respectively (*p* = 0.01). For patients with lung cancer, the administration of a radiation boost improved median OS to 7.3 months compared to a median OS of 4.4 months in the absence of boost; however, the difference was not significant (*p* = 0.1). Significant differences in median OS were observed for patients with gastrointestinal cancer: 2.9 months for patients treated with a boost and 1.3 months in the absence of a boost (*p* = 0.06). For patients with RCC and melanoma, which are both cancers with radio-resistant tumors, the outcome improved after the administration of a radiation boost, compared to WBRT alone, 3.5 *vs.* 3 months, respectively; however, the difference was not significant (*p* = 0.3).

### 2.2. Local Controls and Brain Free-Relapse Controls Benefit from a Radiation Boost

For all 208 patients, a relapse in the brain (local or distant intracerebral failure) occurred in 43 patients (20.7%) after a median interval of 6.9 months (range 1–54 months). Local recurrence was observed for 21 patients (48.9%), regional recurrence was observed for 12 patients (27.9%), and both types of recurrences were observed for 9 patients (20.9%). We did not have this information for one patient.

In patients who received a boost, twenty-three recurrences were observed after a median interval of 8.4 months (range 1–54 months). Fourteen patients (10.6%) experienced local recurrence, 5 patients (3.8%) experienced regional recurrence, and 3 patients (2.3%) experienced both types of recurrence; this information was not available for one patient. In patients who did not receive a boost, twenty recurrences occurred after a median interval of 5.2 months (range 1.2–21.5 months). Local recurrence was observed in 7 patients (9.2%), regional recurrence was observed in 7 patients (9.2%), and both types of recurrence were observed for 6 patients (7.9%) (Table 2).

After WBRT and a radiation boost, the 6-, 12-, 18- and 24-month local control (LC) rates were 92%, 82%, 77.4% and 67%, respectively, and after WBRT alone, they were 81.2%, 75%, 55% and 37.5%, respectively (*p* = 0.03). The 6-, 12-, 18- and 24-month brain control (BC) rates after WBRT and a radiation boost were 88.7%, 75.8%, 69% and 62%, respectively, and after WBRT alone, they were 78.5%, 59%, 44% and 37.7%, respectively (*p* = 0.03). No factors were identified by univariate analysis for local control and brain control (Table 1, Figure 1B,C).

## 3. Discussion

Our study is a large, retrospective analysis of 208 patients from a single institution who had been treated with radiation-based therapies. Radiation therapy was delivered according to RPA class, as established by Gaspar *et al.* in 1200 patients from 3 radiation therapy oncology group (RTOG) trials [10]. In our study, the median survival time was 4.8 months, and this was comparable to the 4.5-month survival time shown in Gaspar *et al.* [10]. The benefit of a radiation boost delivered at the metastatic site remains a controversial subject. Rades *et al.* [11] retrospectively analyzed the data of 416 patients treated with WBRT for multiple brain metastases (BM). Two hundred and fifty seven patients received 30 Gy in 10 fractions of 3 Gy for 2 weeks and were compared to 159 patients treated with 45 Gy in 15 fractions for 3 weeks or 40 Gy in 20 fractions for 4 weeks. They did not demonstrate a significantly better outcome in terms of survival or local control with dose escalation beyond 30 Gy in 10 fractions. However, in another study, Rades *et al.* [12] compared 2 treatment regimens, including surgical resection and WBRT followed or not by a radiation boost to the metastatic site for patients with 1 to 2 BM. Patients who received a boost together with WBRT had a better 1-year OS (66% *vs.* 41%; *p* < 0.001). A radiation boost resulted in a better OS outcome after complete and incomplete surgical resection. However, the brain control and local control results were not statistically significant if surgical resection was incomplete; therefore, further dose escalation and delivery to the metastatic site might be considered.

The EORTC 22952-26001 study included 359 patients with 1 or 3 BM and solid tumors who had been treated with complete surgery or radio-surgery and who had randomly received adjuvant WBRT or were assigned to observation [9]. When the patients’ tumors were not surgically removed, in the WBRT plus boost group, the 2-year local relapse rate was 19% and the brain-relapse rate was 33%, compared to a 33% local relapse rate and a 38% brain relapse rate in our study. Although the local control rates and brain control rates are higher in the EORTC trial, our findings are in accordance with these results. Patients in the EORTC study are more frequently classified as RPA class I due to stable systemic disease, asymptomatic primary tumors and a WHO performance status (PS) of 0 to 2; in our study, all patients were RPA class II. Another difference is the use of radiosurgery for the radiation boost in the EORTC trial, which could explain the superior local control results. The EORTC trial failed to improve overall survival with combined therapy. In our study, the median OS was 5.9 months for patients treated with WBRT and a radiation boost at the metastatic site, and in the EORTC trial, the median survival was 10.7 months in patients treated with WBRT and surgery or radiosurgery. This difference is likely due to the classification of more patients as RPA I in the EORTC trial. Moreover, 64.9% of patients presented an uncontrolled primary tumor in our study, whereas all patients had stable systemic disease or an asymptomatic primary tumor in the EORTC trial. In the RTOG 9508 randomized trial, Andrews *et al.* [13] compared WBRT to WBRT followed by stereotactic radiosurgery boost in patients with 1 to 3 BM. Patients with multiple metastases treated with WBRT and radiosurgery boost or with WBRT alone had median survival times of 5.8 and 6.7 months, respectively (*p* = 0.9). Our findings are in accordance with these results because the median survival time for patients who had been treated with WBRT and radiation boost at the metastatic site was 5.9 months.

In our study, neurological death occurred in 8% of patients who received WBRT and a boost and in 18% of patients treated with WBRT alone. Because this difference was not significant, the difference in OS could not be explained by cranial disease. Moreover, there was a significant difference in the control of primary tumors between the two groups of patients (*p* = 0.04) that could explain the improved OS for patients treated with WBRT and a boost compared to WBRT alone; this was also highlighted by a lowered incidence of extracranial disease. The control of primary tumor may be a prognostic factor of overall and distant free survival for these patients, this would confirm the conclusions of several retrospective studies before [3–5,10]. Moreover, patients with breast cancer and good performance status would have a clinically and statistically significant benefit. Our results suggest that dose escalation may result in an improved outcome in selected patients even if not thought suitable for radiosurgery or surgery alone. A radiation boost delivered with 3D conformal radiotherapy could be applicable to all radiotherapy departments where radiosurgery is not available, increasing access of this treatment to a wider group of patients.

## 4. Materials and Methods

### 4.1. Study Design and Patient Population

The present study was a single institutional, retrospective analysis of a database of 208 patients treated for BM between September 2005 and December 2010 at the Paul Strauss Cancer Center, Strasbourg, France. The method of the study was approved by the institutional review board. The data from RPA II patients with 1 to 2 unresectable BM who had been exposed to different therapies were analysed. Two hundred and eight patients were analysed; among these patients, 132 patients (63.5%) received a radiation boost at the metastatic site, and 76 patients (36.5%) did not receive a boost. The patient characteristics are summarized in Table 3. All patients underwent a CT-scan for delineation, and a customized plastic mask was made to improve set-up. The median interval between BM diagnosis and WBRT was 39 days (1–1005 days). WBRT was performed in patients using two lateral fields from a 6 MV linear accelerator. After 3-dimensional treatment planning, the radiation boost on the operative site was delivered with a 6 or 25 MV linear accelerator using 2 or 3 fields. A total of 30 Gy (10 fractions of 3 Gy) was administered 5 times per week to the whole brain, and a radiation boost was applied at the operative site; a total of 9 Gy (3 fractions of 3 Gy) was administered 3 consecutive times per week. Unfortunately, no toxicity data was available, because this information was not systematically noted, whereas the cause of death could be obtained.

### 4.2. Statistical Analysis

The primary endpoint was defined as the overall survival from the last day of radiotherapy. The secondary endpoints used were local control of the treated lesion and brain relapse-free survival, defined as regional relapse and/or distant intracerebral failure. The time to endpoint was measured from the date of the completion of radiotherapy. All surviving patients at the time of analysis were censored at the date of their last follow-up. Using the Kaplan-Meier method, survival probabilities were calculated for subgroups as defined by the BM treatment type: WBRT alone and WBRT with boost. Seven potential prognostic factors were evaluated: gender, Karnofsky Performance Status, primary tumor type, presence of extracranial metastases (ECM), number of ECM, control of primary tumor, and interval between BM diagnosis and treatment (median: 39 days). All *p* values < 0.05 were considered statistically significant.

## 5. Conclusions

For RPA II patients with 1 or 2 unresectable BM, WBRT treatment and an additional radiation boost delivered with 3D conformal radiotherapy improves local and brain metastatic control rates significantly as well as overall survival.

## Figures and Tables

**Table 1.** Local control and brain control results, as well as overall survival of recursive partitioning analysis (RPA) II patients with 1 or 2 brain metastases (BM) treated with whole brain radiation therapy (WBRT) and with or without boost.

Treatment group	At 6 months (%)	At 12 months (%)	At 18 months (%)	At 24 months (%)	*p*
Local control					

Boost	92	82	77.4	67	0.03
No boost	81.2	75	55	37.5	

Brain control					

Boost	88.7	75.8	69	62	0.03
No boost	78.5	59	44	37.7	

Overall survival					

Boost	48.5	25	17.4	10.6	0.03
No boost	34	22.4	12	3.2	

**Table 2.** Brain, local and regional recurrences organized by radiation boost. *N*, patient’s number; *n*, number of recurrences.

Treatment group	Brain recurrence *n*	Local recurrence *n* (% of N)	Regional recurrence *n* (% of N)	Both *n* (% of N)	Unknown *n* (% of N)
Boost (*N* = 132)	23	14 (10.6)	5 (3.8)	3 (2.3)	1 (0.75)
No boost (*N* = 76)	20	7 (9.2)	7 (9.2)	6 (7.9)	0
Total (*N* = 208)	43	21 (48.8)	12 (27.9)	9 (20.9)	1 (2.3)

**Table 3.** Patient characteristics. KPS, Karnofsky Performance Status; BM, brain metastases; ECM, extracranial metastases; GI, gastrointestinal; RCC, renal cell cancer; N, patient’s number.

Characteristic	Value			*p*

	Entire series (*n* = 208) *N* (%)	Boost (*n* = 132) *N* (%)	No boost (*n* = 76) *N* (%)	
Age (year)				0.33
median	64 (20.9–86.2)	63.4 (21.2–86.2)	65.2 (20.9–85.7)	

Gender				0.67
Male	153 (73.6)	96 (72.7)	57 (75.0)	
Female	55 (26.4)	36 (27.3)	19 (25.0)	

KPS				0.79
70–80	143 (68.8)	91 (68.9)	52 (68.4)	
90–100	65 (31.2)	41 (31.1)	24 (31.6)	

BM				0.48
1	122 (58.7)	90 (68.2)	32 (42.1)	
2	86 (41.3)	42 (31.8)	44 (57.9)	

ECM				0.9
Yes	169 (81.2)	106 (80.3)	63 (82.9)	
No	39 (18.8)	26 (19.7)	13 (17.1)	

Number of ECM				0.35
1	67 (39.6)	41 (38.7)	26 (41.3)	
≥2	102 (60.4)	65 (61.3)	37 (58.7)	

Control of primary tumor				0.04
Yes	73 (35.1)	56 (42.4)	17 (22.4)	
No	135 (64.9)	76 (57.6)	59 (77.6)	

Neurological symptoms				0.9
Yes	112 (53.8)	74 (56.1)	38 (50.0)	
No	96 (46.2)	58 (43.9)	38 (50.0)	

Site of primary tumor				–
Lung	137 (65.9)	85 (64.4)	52 (68.4)	
Breast	18 (8.6)	13 (9.8)	5 (6.6)	

Melanoma	10 (4.8)	4 (3.0)	6 (7.9)	
GI	16 (7.7)	12 (9.2)	4 (5.3)	
RCC	7 (3.4)	5 (3.8)	2 (2.6)	

Other	20 (9.6)	13 (9.8)	7 (9.2)	
